# Vagus nerve stimulation as a potential adjuvant to behavioral therapy for autism and other neurodevelopmental disorders

**DOI:** 10.1186/s11689-017-9203-z

**Published:** 2017-07-04

**Authors:** Crystal T. Engineer, Seth A. Hays, Michael P. Kilgard

**Affiliations:** 1 Texas Biomedical Device Center, The University of Texas at Dallas, 800 West Campbell Road BSB11, Richardson, TX 75080 USA; 2 School of Behavioral and Brain Sciences, The University of Texas at Dallas, 800 West Campbell Road BSB11, Richardson, TX 75080 USA; 3 Erik Jonsson School of Engineering and Computer Science, The University of Texas at Dallas, 800 West Campbell Road BSB11, Richardson, TX 75080 USA

**Keywords:** Vagal nerve, Plasticity, Cortex, Cortical reorganization, Autism

## Abstract

**Background:**

Many children with autism and other neurodevelopmental disorders undergo expensive, time-consuming behavioral interventions that often yield only modest improvements. The development of adjunctive interventions that can increase the benefit of rehabilitation therapies is essential in order to improve the lives of individuals with neurodevelopmental disorders.

**Main text:**

Vagus nerve stimulation (VNS) is an FDA approved therapy that is safe and effective in reducing seizure frequency and duration in individuals with epilepsy. Individuals with neurodevelopmental disorders often exhibit decreased vagal tone, and studies indicate that VNS can be used to overcome an insufficient vagal response. Multiple studies have also documented significant improvements in quality of life after VNS therapy in individuals with neurodevelopmental disorders. Moreover, recent findings indicate that VNS significantly enhances the benefits of rehabilitative training in animal models and patients, leading to greater recovery in a variety of neurological diseases. Here, we review these findings and provide a discussion of how VNS paired with rehabilitation may yield benefits in the context of neurodevelopmental disorders.

**Conclusions:**

VNS paired with behavioral therapy may represent a potential new approach to enhance rehabilitation that could significantly improve the outcomes of individuals with neurodevelopmental disorders.

## Background

### Current therapies for autism spectrum disorders

Children with autism and other neurodevelopmental disorders often exhibit a myriad of symptoms, including social communication deficits, restricted or repetitive behaviors, epilepsy, language impairment, anxiety, developmental delays, and sensory processing problems. These symptoms arise in part from alterations in neural function. Intensive behavioral therapies can be successfully used to improve behavioral outcomes in these individuals by promoting adaptive plasticity in dysfunctional neural circuits [1–4].

A proportion of children with autism who undergo intensive interventions experience significant gains in intelligence, communication, and social skills, highlighting the importance of behavioral therapies [5–7]. However, these interventions are very time-consuming (more than 20 h/week), expensive (more than $30,000 per year per child), and a substantial proportion of children fail to benefit, thereby limiting the utility of these strategies [8–11]. The development of adjunctive techniques to enhance neuroplasticity and subsequently increase the benefit of rehabilitation therapies holds promise to improve the lives of individuals with neurodevelopmental disorders.

### Vagus nerve stimulation therapy

Vagus nerve stimulation (VNS) has emerged as a potential adjunctive therapy for individuals with autism. VNS is an FDA-approved therapy used to decrease the severity of treatment-resistant epilepsy and depression. An implanted pulse generator delivers intermittent trains of electrical stimulation via an electrode cuff placed around the left cervical branch of the vagus nerve. Numerous studies have documented that VNS therapy is safe and can be delivered to adults, as well as children as young as 6 months of age, with minimal adverse effects [12–15]. Side effects, such as coughing and hoarseness, are usually mild and temporary [16, 17]. In addition to stimulation via an implanted device, non-invasive stimulation of either the cervical or auricular branches of the vagus nerve also may be particularly useful in patients with neurodevelopmental disorders, although considerably more testing is required to demonstrate the efficacy of non-invasive stimulation paradigms [18]. As the preponderance of studies utilize invasive VNS, this review will focus on this delivery method.

A substantial portion of epilepsy patients experience a significant reduction in both seizure frequency and severity, and it has been well-documented that responder rates increase over time [13, 19–21]. Approximately 30–40% of patients experience a ≥50% decrease in seizure frequency following 3–6 months of VNS therapy, while more than 60% of patients experience a ≥50% decrease in seizure frequency following 24 months of therapy [13, 21–23]. In addition to a reduction in seizures, significant quality of life improvements are often noted, such as increased alertness, communication, independence, memory, mood, and sleep [12, 16, 23–33]. Unlike antiepileptic drugs, VNS therapy has no reported negative effects on cognition [27]. Rather, a number of animal studies suggest that VNS may improve memory [34, 35]. In addition, VNS has antidepressant effects and enhances neurocognitive function, including executive functions and language, in patients with treatment-resistant depression [36, 37]. Moreover, a study of individuals with epilepsy documented that VNS delivered following the reading of paragraphs significantly enhanced word recognition memory [33]. Similarly, non-verbal children with epilepsy have been reported to speak their first words following VNS therapy [30]. Interestingly, these quality of life improvements are often documented to be independent of changes in seizure frequency, suggesting that multiple pathways may be engaged by VNS to yield beneficial effects [27, 31, 38].

## Main text

### VNS therapy in ASD and other neurodevelopmental disorders

A number of lines of evidence indicate that VNS may provide some symptomatic relief for individuals with autism. In many autism spectrum disorders (ASD), the parasympathetic system is dysregulated and reduced vagal tone is frequently observed [39–43]. Diminished vagal activity has been associated with both autistic behaviors and language impairment [42, 43], while higher vagal activity has been shown to predict better communication outcomes later in life [44]. VNS therapy has previously been shown to overcome an insufficient vagal response [45], suggesting that VNS therapy may be beneficial in neurodevelopmental disorders with altered parasympathetic activity.

Studies using VNS in children with both epilepsy and an autism spectrum disorder have also yielded positive results. VNS reduces seizure frequency and improves quality of life in individuals with ASD [38, 46–51]. In the largest study to date of VNS therapy in individuals with ASD, seizure reduction was similar between individuals with and without autism [46]. After 12 months of VNS therapy, 56% of individuals without autism experienced a ≥50% reduction in seizures, while 62% of individuals with autism experienced a ≥50% reduction in seizures. Individuals with and without autism exhibited similar improvements in alertness, verbal communication, memory, and school/professional achievements. However, individuals with autism also experienced a significantly greater improvement in mood following 12 months of VNS therapy compared to individuals without autism.

Another large study documented the outcomes of VNS therapy in individuals with Landau-Kleffner syndrome (acquired epileptiform aphasia) and individuals with autism [48]. Both groups experienced improvements in seizure frequency and quality of life. Among individuals with autism, a majority of patients experienced a ≥50% reduction in seizures and improvements in alertness, achievement, and mood.

A handful of smaller studies and case studies have also documented the positive outcomes of VNS therapy in individuals with neurodevelopmental disorders. In one striking study in girls with Rett syndrome and medically refractory epilepsy, 86% of girls had a ≥50% reduction in seizure frequency following 1 year of VNS therapy, and 100% of girls exhibited increased alertness [50]. A case report described an individual with Asperger syndrome and epilepsy who experienced a 69% decrease in the number of seizures as well as an 83% decrease in seizure duration following 6 months of VNS therapy [49]. Additionally, the patient had significant improvements in mood and achievements, and a reduction of aggressive outbursts. In a separate study, children with hypothalamic hamartomas, epilepsy, and severe autistic behaviors each experienced significant behavioral improvements following VNS therapy that did not correlate with seizure control [47].

Despite the generally positive evidence of VNS on epilepsy in the context of neurodevelopmental disorders, two small studies reported no improvement in seizure control. A case report described an individual with ASD and epilepsy who experienced an initial decrease in seizure severity and duration that only lasted 6 months [38]. Despite the lack of seizure control, over the subsequent 6 months, the patient had a significant decrease in aggressive behaviors and stereotype and a significantly increased ability to follow directions. A separate study observed no improvement in seizure frequency in children with ASD despite mild improvement in social abilities [52]. Overall, responder rates are high considering that all patients who underwent VNS therapy had medically refractory epilepsy that was resistant to pharmacological treatment. However, there is still room for optimization of VNS therapy for epilepsy control in individuals with neurodevelopmental disorders.

### VNS paired with rehabilitation

There is growing preclinical and clinical evidence that pairing bursts of VNS with specific movements or sensory events can improve rehabilitation of stroke, tinnitus, traumatic brain injury, spinal cord injury, and post-traumatic stress disorder [53]. In contrast to the open-loop delivery of 30-s long stimulation trains for epilepsy, emerging applications of VNS use short 0.5-s trains delivered coincident with specific events, such as hearing a sound or moving the hand, during a training or rehabilitative paradigm. This paradigm is based on VNS-dependent activation of plasticity-enabling neuromodulatory circuits to reinforce the neural activity associated with rehabilitation [53]. VNS pairing therapy can reorganize the brain in a manner that is both highly specific to the paired experience and long lasting. For example, pairing VNS with the presentation of a tone causes neurons in the auditory cortex to shift their preference to match the pitch of the paired tone [54, 55]. Pairing VNS with the presentation of fast or slow trains of tones increases or decreases the ability of auditory cortex to respond to rapid sounds [56]. Pairing VNS with the presentation of specific consonant sounds specifically increases the auditory cortex response strength to the paired sounds and decreases the response latency [57]. VNS pairing can also drive specific neural plasticity in motor cortex. Pairing VNS with distal forelimb movement during motor training increases the representation of the distal forelimb musculature in the motor cortex, while pairing VNS with proximal forelimb movement increases the cortical representation of the proximal forelimb [58, 59]. Together, these findings indicate the flexibility of pairing VNS with disparate training paradigms to drive specific neural plasticity.

Based on this robust, specific enhancement of plasticity with training, several studies have tested whether VNS paired with rehabilitation may enhance the benefits of rehabilitation and support recovery in the context of neurological disease. Indeed, a number of studies in animal models and patients demonstrate that pairing VNS with sensory or motor therapy can dramatically improve functional outcomes. Pairing VNS with tones eliminated tinnitus-related behavior and normalized neural activity in a rat model of tinnitus [54]. When the same therapy was delivered in chronic tinnitus patients, it provided long-lasting improvement in tinnitus intensity and tinnitus distress, highlighting the translational potential of paired VNS [60, 61]. Additional studies provide evidence that paired VNS may be useful to treat motor dysfunction resulting from neurological damage. Pairing VNS with physical rehabilitation therapy improves recovery in multiple mechanistically distinct rat models of brain injury, including ischemic stroke, intracerebral hemorrhage, and traumatic brain injury [62–66]. Other VNS paradigms highlight the potential for VNS to modulate ischemia and inflammation [67, 68]. A recent pilot in chronic stroke patients reinforces the clinical potential of VNS therapy. VNS paired with physical rehabilitation yields threefold greater improvements in upper limb function compared to rehabilitation alone [69]. Moreover, preclinical studies provide evidence that VNS paired with cognitive therapy may yield benefits. Pairing VNS with extinction therapy influences expression and activation of proteins associated with synaptic plasticity, including CaMKII, Arc, and GluN2B [70]. Consistent with these changes in plasticity-associated proteins, VNS paired with extinction therapy accelerates the extinction of a conditioned fear response in an animal model of anxiety [45, 71]. These studies demonstrate the flexibility of VNS paired across a range of sensory, motor, and cognitive therapies and highlight its potential as a platform technology.

### Delivery of VNS therapy for neurodevelopmental disorders

We propose that VNS could be paired with a variety of rehabilitative therapies targeting distinct dysfunctions to enhance adaptive neuroplasticity and improve outcomes in individuals with neurodevelopmental disorders. VNS could be incorporated into sessions of speech therapy, occupational therapy, physical therapy, or autism therapy. For VNS paired speech therapy sessions, a short burst of VNS could be delivered by a speech therapist with each attempt to speak a sound. During occupational therapy sessions, VNS could occur with each attempted movement, similar to the implementation used in stroke patients during physical rehabilitation [69]. A key benefit of VNS therapy is the ability for stimulation to be paired with well-established, existing rehabilitative interventions. The demonstrated flexibility of VNS to enhance a variety of rehabilitative training regimens holds potential to treat the many facets of dysfunction that accompany neurodevelopmental disorders, but future preclinical studies and clinical trials are necessary in order to evaluate and optimize the potential of paired VNS therapies.

### Therapeutic mechanisms of VNS

VNS therapy is believed to enhance the benefits of rehabilitation by engaging the plasticity-enabling cholinergic and noradrenergic neuromodulatory systems during training. Stimulation of the vagus nerve drives robust, phasic neural activity in the locus coeruleus, the primary source of norepinephrine in the central nervous system (CNS) [72]. Consistent with VNS-dependent engagement of the noradrenergic system, VNS increases norepinephrine levels in the hippocampus and cortex [73–75]. Additionally, VNS significantly increases levels of brain-derived neurotrophic factor (BDNF); a neurotrophin strongly linked to neural plasticity and dysregulated in autistic individuals [76–79]. Pointing to the requirement of these neuromodulatory systems, a reduction in either noradrenergic or cholinergic transmission blocks the effects of VNS in the central nervous system. Lesions of the locus coeruleus prevent VNS-dependent reduction in seizures, and antagonism of β-adrenergic receptors blocks VNS-dependent plasticity [80, 81]. Similarly, depletion of acetylcholine prevents VNS-dependent enhancement of plasticity in motor cortex [59]. Recent clinical studies provide additional indirect evidence of the importance of these neuromodulatory systems. In a study evaluating VNS paired with tones to treat chronic tinnitus, the majority of patients that failed to benefit from VNS were on medications that interfered with neuromodulatory transmission [60]. Together, these findings highlight the importance of the cholinergic and noradrenergic systems in VNS therapy [60]. Several neurodevelopmental disorders, including Down syndrome, Rett syndrome, and Fragile X syndrome display alterations in the function of these neuromodulatory systems [82–88]. As a result of these baseline changes, VNS-dependent engagement of neuromodulatory systems may be altered in these individuals, which could in turn reduce efficacy. Alternatively, the ability to provide repeated, robust activation of these neuromodulatory systems using VNS at specific times during training exercises may promote substantial enhancement of plasticity and increase the benefits of rehabilitation.

A key component in VNS-dependent enhancement of plasticity is the close temporal association of stimulation and specific events during rehabilitation. Engagement of neuromodulatory circuits by short trains of VNS provides a precisely timed, phasic release of neuromodulators that serves to reinforce neural circuits activated during rehabilitation [72]. This temporal association between VNS and rehabilitation provides the specificity to target plasticity to particular neural circuits. Indeed, in a study evaluating VNS paired with rehabilitation to promote recovery after spinal cord injury, VNS enhanced network plasticity and synaptic connectivity specifically in neural circuits associated with control of the rehabilitated muscle groups [89]. Perhaps even more strikingly, a study in a rat model of tinnitus demonstrated that repeatedly pairing VNS with a 9 kHz tone increased the neural response to the paired tone but not to an interleaved 4 kHz tone presented within seconds of stimulation [54]. Consistent with the concept of precise timing mediating reinforcement, a number of studies demonstrate that an equivalent amount of VNS that is not paired with rehabilitative training fails to enhance the benefits of rehabilitation [63, 90]. The requirement for temporal association between neural activity and VNS is consistent with studies evaluating the timing of neuromodulator release on synaptic plasticity and supports the role of phasic activation of the cholinergic and noradrenergic systems in VNS-dependent benefits [91, 92]. The absence of benefits when VNS is not paired with rehabilitation indicates that VNS is likely acting to improve function by engaging neural plasticity, rather than through generalized temporally independent processes such as reduction of inflammation or neurogenesis.

**Fig. 1:**
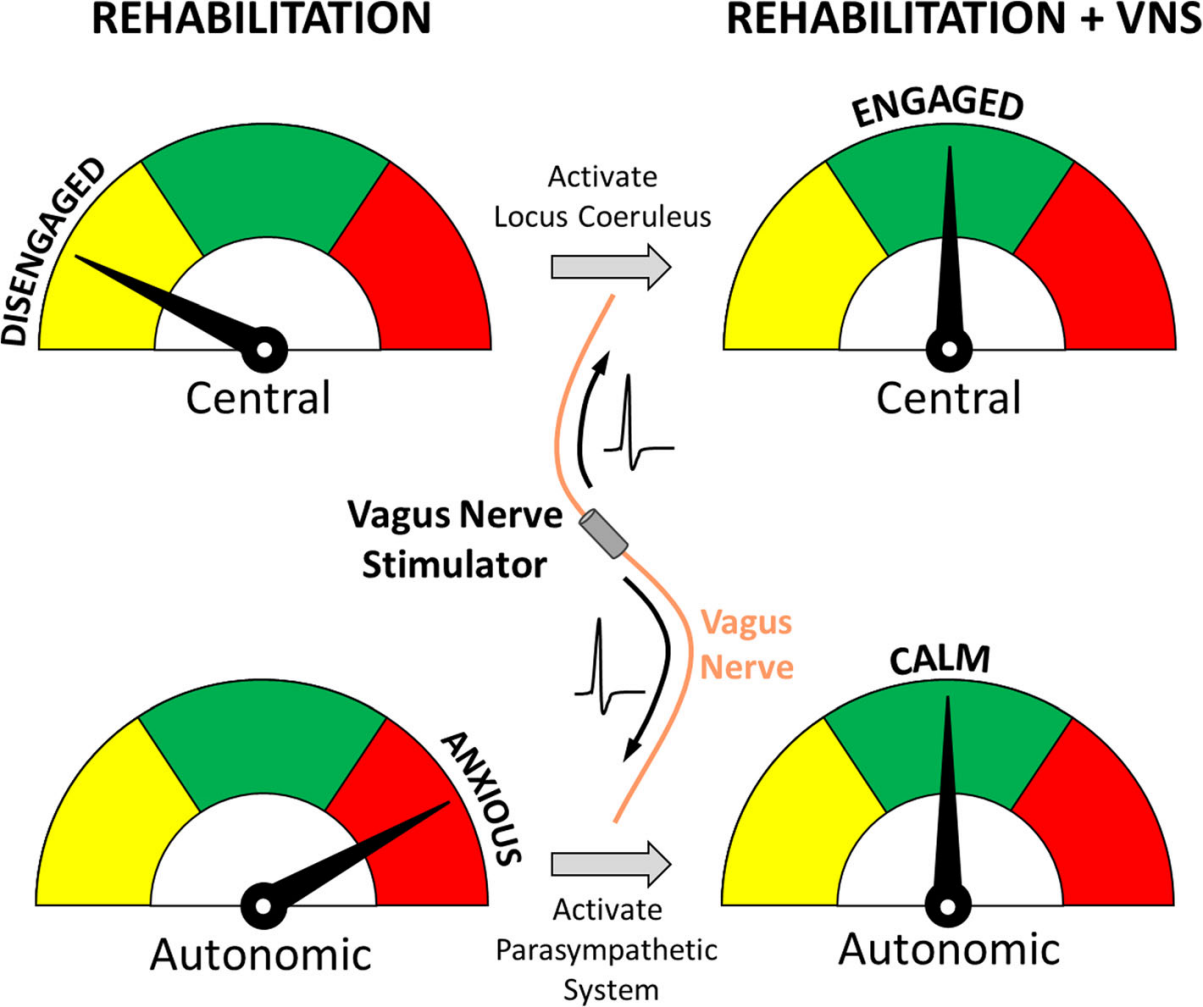
Dual action of VNS. Stimulation of the vagus nerve at the cervical level drives nerve action potentials to activate afferent projections to CNS neuromodulatory centers (*top*) in conjunction with activation of parasympathetic efferent fibers (*bottom*). This unique dual action of VNS provides both a neural plasticity-reinforcing stimulus through afferent activation and a calming parasympathetic input through efferent activation

In addition to activation of neuromodulatory centers in the central nervous system, VNS provides concomitant activation of the parasympathetic branch of the autonomic nervous system. The vagus nerve consists of a major portion of the descending parasympathetic fibers innervating the viscera. As a result, stimulation of the cervical vagus nerve activates these parasympathetic efferent fibers in conjunction with activation of afferent projections to CNS neuromodulatory centers (Fig. 1). Parasympathetic activation is associated with “rest-and-digest” physiological responses, and the vagus nerve carries information that mediates the transition to calm behavioral states [93, 94]. Consistent with this, a study in patients demonstrates that VNS provides a reduction in measures of anxiety [95]. The dual action of VNS in providing both a neural plasticity reinforcing stimulus through activation of ascending pathways and a calming parasympathetic input through activation of descending pathways is a unique property that may confer benefits not currently available with pharmacological interventions [96]. Drugs that activate neuromodulatory systems can enhance learning but often promote anxiety, opposing parasympathetic activation. Alternatively, anxiolytic drugs tend to interfere with plasticity and blunt the benefits of rehabilitation. Moreover, VNS-dependent parasympathetic activation may overcome altered vagal tone and autonomic dysregulation associated with a number of neurodevelopmental disorders [40–44]. At present, the ability of VNS to provide parasympathetic benefits in the context of neurodevelopmental disorders is an emerging field of study [38, 49, 51]; however, given the potential benefits of VNS, further evaluation seems warranted.

One critical step in the development of VNS-based therapy for individuals with neurodevelopmental disorders will be defining the most appropriate stimulation paradigm to confer both epilepsy suppression and enhancement of rehabilitative benefits. Presently, semi-continuous 30 s on and 5 min off cycling stimulation has been used for epilepsy control, whereas short 0.5 s bursts of VNS paired with rehabilitation have been employed for targeted plasticity therapies. To date, no studies have evaluated long 30 s stimulation paradigms for enhancement of rehabilitation or short 0.5 s stimulation for epilepsy control. Clearly, if either stimulation paradigm successfully resulted in both epilepsy control and enhanced rehabilitative benefits, this stimulation paradigm should be employed. Alternatively, a hybrid combination incorporating elements of both stimulation paradigms may be most effective. For instance, individuals may receive only short bursts of VNS paired with specific events during rehabilitative sessions and subsequently receive 30 s on and 5 min off cycling stimulation when not undergoing rehabilitation. In conjunction with the potential influence of altered plasticity and neuromodulatory function, it is clear that significant efforts should be made to develop optimal VNS paradigms to provide maximal benefits for both seizure suppression and enhanced rehabilitation for individuals with neurodevelopmental disorders.

## Conclusions

VNS paired with rehabilitation has emerged as an intervention that significantly improves sensory, motor, and cognitive deficits in a variety of neurological disorders. Here, we suggest that pairing VNS with rehabilitative therapy may represent a potential new approach to enhance current rehabilitative therapies and to improve the functional outcomes of individuals with neurodevelopmental disorders. Significant development of VNS therapy remains, and delivery may be limited by device cost, surgical complications, adverse effects, and the possibility that VNS-paired therapy does not enhance recovery in all patients. Future studies should evaluate both invasive and non-invasive VNS paired with rehabilitative therapies on a range of sensory, motor, and cognitive functions in a variety of neurodevelopmental disorders and focus on identifying optimal stimulation parameters to maximize therapeutic benefits.

## Abbreviations

ASD: Autism spectrum disorder
CNS: Central nervous system
FXS: Fragile X syndrome
VNS: Vagus nerve stimulation
